# 1-(4-Iodo­benz­yl)-3-methyl­pyridinium bis­(benzene-1,2-dithiol­ato)nickelate(III)

**DOI:** 10.1107/S1600536811043522

**Published:** 2011-10-29

**Authors:** Guang-Xiang Liu

**Affiliations:** a School of Biochemical and Environmental Engineering, Nanjing Xiaozhuang University, Nanjing 211171, People’s Republic of China

## Abstract

The asymmetric unit of the title compound, (C_13_H_13_IN)[Ni(C_6_H_4_S_2_)_2_], contains half each of two centrosymmetric anions and a single cation in a general position. In the anions, the Ni^III^ ions are surrounded by four S atoms in a distorted square-planar geometry. In the crystal, the anions exhibit two different packing modes by stacking along the *a* axis in face-to-face and side-by-side fashions. Inter­ionic C—H⋯S hydrogen bonds and π–π stacking inter­actions [centroid–centroid distance = 3.6947 (5) Å] are observed.

## Related literature

For background to the synthesis and properties of metal complexes of dithiol­ato and dithiol­ene ligands, see: Robertson & Cronin (2002 ▶); Kato (2004 ▶); Cassoux (1999 ▶); Canadell (1999 ▶); Akutagawa & Nakamura (2000 ▶); Ren *et al.* (2002 ▶, 2004 ▶, 2008 ▶). For the structure of a related compound, see: Liu *et al.* (2007 ▶).
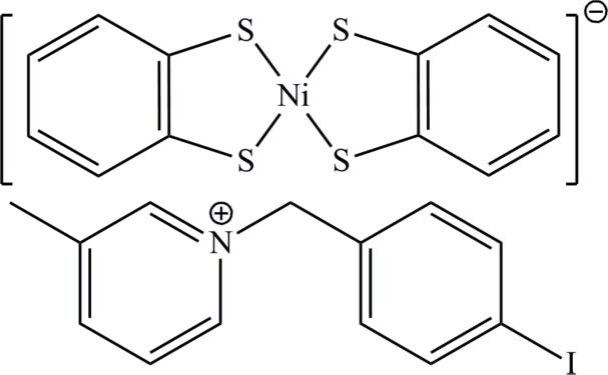


## Experimental

### 

#### Crystal data

(C_13_H_13_IN)[Ni(C_6_H_4_S_2_)_2_]
*M* *_r_* = 649.28
Triclinic, 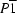

*a* = 7.3222 (14) Å
*b* = 12.267 (2) Å
*c* = 14.628 (3) Å
α = 98.425 (2)°
β = 98.466 (2)°
γ = 96.216 (3)°
*V* = 1274.2 (4) Å^3^
*Z* = 2
Mo *K*α radiation
μ = 2.32 mm^−1^
*T* = 296 K
0.26 × 0.20 × 0.12 mm

#### Data collection

Bruker SMART APEX CCD area-detector diffractometer
Absorption correction: multi-scan (*SADABS*; Bruker, 2000 ▶) *T*_min_ = 0.584, *T*_max_ = 0.769
6282 measured reflections
4392 independent reflections
3397 reflections with *I* > 2σ(*I*)
*R*_int_ = 0.030

#### Refinement

*R*[*F* ^2^ > 2σ(*F* ^2^)] = 0.056
*wR*(*F* ^2^) = 0.196
*S* = 1.09
4392 reflections
293 parameters
H-atom parameters constrained
Δρ_max_ = 1.52 e Å^−3^
Δρ_min_ = −1.91 e Å^−3^

### 

Data collection: *SMART* (Bruker, 2000 ▶); cell refinement: *SAINT* (Bruker, 2000 ▶); data reduction: *SAINT*; program(s) used to solve structure: *SHELXS97* (Sheldrick, 2008 ▶); program(s) used to refine structure: *SHELXL97* (Sheldrick, 2008 ▶); molecular graphics: *SHELXTL* (Sheldrick, 2008 ▶); software used to prepare material for publication: *SHELXTL*.

## Supplementary Material

Crystal structure: contains datablock(s) I, global. DOI: 10.1107/S1600536811043522/rz2652sup1.cif

Structure factors: contains datablock(s) I. DOI: 10.1107/S1600536811043522/rz2652Isup2.hkl

Additional supplementary materials:  crystallographic information; 3D view; checkCIF report

## Figures and Tables

**Table 1:** Hydrogen-bond geometry (Å, °)

*D*—H⋯*A*	*D*—H	H⋯*A*	*D*⋯*A*	*D*—H⋯*A*
C19—H19*B*⋯S2^i^	0.97	2.81	3.613 (7)	141
C20—H20⋯S4^ii^	0.93	2.86	3.580 (8)	135

Symmetry codes: (i) 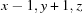; (ii) 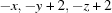.

**Fig. 1.**
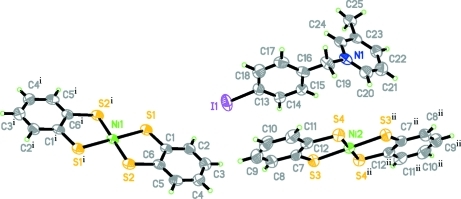
The molecular structure of the title compound, with displacement ellipsoids drawn at the 30% probabilility level. Symmetry codes: (i) 1-x, -y, 1-z; (ii) 1-x, 2-y, 2-z.

**Fig. 2.**
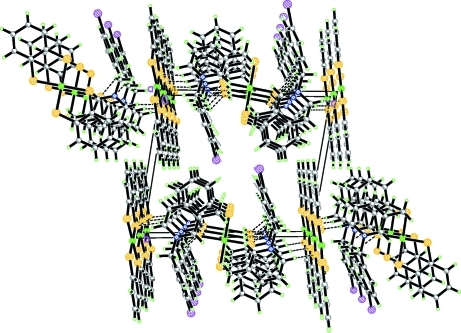
Packing diagram of the title compound viewed along the a axis. Hydrogen bonds are shown as dashed lines.

## supplementary crystallographic information

### Comment

Metal complexes of 1,2-dithiolate ligands have been intensively studied because of their novel properties and applications in the areas of molecular conductivity, magnetic materials, nonlinear optics, and others (Robertson & Cronin, 2002; Kato, 2004). Over the last decade, a large number of new dithiolene ligands and metal complexes have been prepared in an effort to obtain novel and advanced material, whose molecular arrangement can be sensitively affected by strong and directional noncovalent interactions (Cassoux, 1999; Canadell, 1999; Akutagawa & Nakamura, 2000). Although the closed-shell cations make no contribution to the conductivity and magnetism, their size and shapes play a predominant role in influencing the crystal structure and, consequently, in altering the electronic and magnetic properties. Recently, using benzylpyridinium derivatives ([RBzPy]^+^) as counter cation of [*M*(mnt)_2_]^-^ (*M* = Ni, Pd, and Pt; mnt = maleonitriledithiolate), a series of ion-pair complexes with segregated columnar stacks of cations and anions have been reported (Ren *et al.*, 2002, 2008). The quasi-one-dimensional magnetic nature of these complexes was attributed to intermolecular π orbital interactions within the anionic columns. Furthermore, for some complexes, spin-Peierls-like transition was observed (Ren *et al.*, 2004). More presently, we are devoted our research interesting on the molecular magnets self-assembled from the [Ni(bdt)_2_]^-^ ion (bdt = benzene-1,2-dithiolato) due to its molecular and electronic structure resembling the [Ni(mnt)_2_]^-^ ion, which is expected to obtain new series of molecular magnets with peculiar magnetic phase transition *via* incorporating benzylpyridinium derivatives into the [Ni(bdt)_2_]^-^ spin system. The synthesis and crystal structure of the title compound, a new ion-pair complex, is reported herein.

As shown in Fig. 1, the asymmetric unit of the title complex contains two independent halves of centrosymmetric [Ni(bdt)_2_]^-^ anions and one [IBzPyCH_3_]^+^ cation. The nickel atoms are each surrounded by four sulfur atoms in a square-planar geometry. As for the Ni1-containing unit, the Ni1—S1 and Ni1—S2 distances are 2.1470 (16) and 2.1562 (16) Å, respectively. These values are in agreement with those found in the related [Ni(bdt)_2_]^-^ complex reported recently (Liu *et al.*, 2007). The S—Ni—S bond angle is 91.59 (6)°, which is slightly larger than that observed in the complex with substituent groups on benzene rings (Liu *et al.*, 2007). There exists a dihedral angle of 6.92 (6)° between the planes through C1–C6/S1/S2 and Ni1/S1/S2, so the five-membered ring adopts an envelope conformation and the Ni1 atom deviates by 0.1808 (2) Å from the C_6_S_2_ plane. In the Ni2-containing unit, the Ni—S bonds cover the range from 2.1447 (15) to 2.1504 (17) Å and the S—Ni—S bond angle 91.79 (6)°. The Ni2 atom deviates by 0.0245 (2) Å from the C7–C12/S3/S4 plane and the angle between the C_6_S_2_ and Ni2/S3/S4 planes is 0.98 (8)°. The two C_6_S_2_ planes are roughly perpendicular to each other with a dihedral angle of 73.67 (7)°. In the [IBzPyCH_3_]^+^ cation, the dihedral angles between the phenyl and pyridine rings is 86.7 (2)°. The molecule packings of two anionic units differ from each other (Fig. 2). The Ni1-containing anions stack in face-to-face fashion along the *a* axis with an alternating arrangement of the [Ni(bdt)_2_]^-^anions and [IBzPyCH_3_]^+^ cations, so that the pyridine ring of the cation overlaps the phenyl ring of the anion with a centroid-to-centroid distance of 3.6947 (5) Å. Conversely, the Ni2-containing anions stack in side-by-side fashion in which the anions are uniformly spaced to form one-dimensional chains along the *a* axis. For both stacking modes the Ni···Ni separation is 7.3223 (14) Å. The shortest separation between Ni1-containing and Ni2-containing anions is 7.3140 (15) Å. The anions and cation are linked *via* C—H···S H-bonding interactions consolidating the crystal structure (Table 1).

### Experimental

Under argon atmosphere at room temperature, benzene-1,2-dithiol (284 mg, 2 mmol) was added to a solution of sodium metal (92 mg, 4 mmol) in absolute methanol (25 ml. A solution of NiCl_2_.6H_2_O (240 mg, 1 mmol) in methanol was then added, resulting in the formation of a muddy red-brown colour. Following this, 1-(4-iodobenzyl)-3-methylpyridinium bromide (780 mg, 2 mmol) was added, the mixture allowed to stand with stirring for 1 h and then stirred for additional 24 h in air. The colour of the mixture gradually turned green, indicating oxidation from a dianionic species to the more stable monoanionic form. The precipitate was washed with absolute methanol and ether and then dried. The crude product was recrystallized twice from methylene chloride to give dark green crystals suitable for X-ray analysis in ~52% yield.

### Refinement

H atoms were positioned geometrically, with C—H = 0.93, 0.97 and 0.96 Å for aromatic, methylene and methyl H atoms, respectively, and constrained to ride on their parent atoms, with *U*_iso_(H) = x *U*_eq_(C), where x = 1.5 for methyl H and x = 1.2 for all other H atoms. The highest peak (1.52 e\&A^-3^) and the deepest hole (-1.91 e\&A^-3^) are located 1.00 and 0.96 Å, respectively, from I1.

### Crystal data

**Table:**

(C_13_H_13_IN)[Ni(C_6_H_4_S_2_)_2_]	*Z* = 2
*M**_r_* = 649.28	*F*(000) = 646
Triclinic, *P*1	*D*_x_ = 1.692 Mg m^−^^3^
Hall symbol: -P 1	Mo *K*α radiation, λ = 0.71073 Å
*a* = 7.3222 (14) Å	Cell parameters from 2848 reflections
*b* = 12.267 (2) Å	θ = 2.4–27.0°
*c* = 14.628 (3) Å	µ = 2.32 mm^−^^1^
α = 98.425 (2)°	*T* = 296 K
β = 98.466 (2)°	Block, dark green
γ = 96.216 (3)°	0.26 × 0.20 × 0.12 mm
*V* = 1274.2 (4) Å^3^	

### Data collection

**Table:**

Bruker SMART APEX CCD area-detector diffractometer	4392 independent reflections
Radiation source: sealed tube	3397 reflections with *I* > 2σ(*I*)
graphite	*R*_int_ = 0.030
φ and ω scans	θ_max_ = 25.0°, θ_min_ = 1.4°
Absorption correction: multi-scan (*SADABS*; Bruker, 2000)	*h* = −8→8
*T*_min_ = 0.584, *T*_max_ = 0.769	*k* = −14→13
6282 measured reflections	*l* = −17→17

### Refinement

**Table:**

Refinement on *F*^2^	Primary atom site location: structure-invariant direct methods
Least-squares matrix: full	Secondary atom site location: difference Fourier map
*R*[*F*^2^ > 2σ(*F*^2^)] = 0.056	Hydrogen site location: inferred from neighbouring sites
*wR*(*F*^2^) = 0.196	H-atom parameters constrained
*S* = 1.09	*w* = 1/[σ^2^(*F*_o_^2^) + (0.1231*P*)^2^ + 0.2746*P*] where *P* = (*F*_o_^2^ + 2*F*_c_^2^)/3
4392 reflections	(Δ/σ)_max_ = 0.001
293 parameters	Δρ_max_ = 1.52 e Å^−^^3^
0 restraints	Δρ_min_ = −1.91 e Å^−^^3^

### Special details

**Table:**

Geometry. All e.s.d.'s (except the e.s.d. in the dihedral angle between two l.s. planes) are estimated using the full covariance matrix. The cell e.s.d.'s are taken into account individually in the estimation of e.s.d.'s in distances, angles and torsion angles; correlations between e.s.d.'s in cell parameters are only used when they are defined by crystal symmetry. An approximate (isotropic) treatment of cell e.s.d.'s is used for estimating e.s.d.'s involving l.s. planes.
Refinement. Refinement of *F*^2^ against ALL reflections. The weighted *R*-factor *wR* and goodness of fit *S* are based on *F*^2^, conventional *R*-factors *R* are based on *F*, with *F* set to zero for negative *F*^2^. The threshold expression of *F*^2^ > σ(*F*^2^) is used only for calculating *R*-factors(gt) *etc*. and is not relevant to the choice of reflections for refinement. *R*-factors based on *F*^2^ are statistically about twice as large as those based on *F*, and *R*- factors based on ALL data will be even larger.

### Fractional atomic coordinates and isotropic or equivalent isotropic displacement parameters (Å^2^)

**Table:**

	*x*	*y*	*z*	*U*_iso_*/*U*_eq_	
Ni1	0.5000	0.0000	0.5000	0.0571 (3)	
Ni2	0.5000	1.0000	1.0000	0.0538 (3)	
S1	0.5791 (2)	0.17354 (14)	0.49920 (11)	0.0667 (4)	
S2	0.4882 (2)	0.03099 (14)	0.64783 (11)	0.0659 (4)	
S3	0.6151 (2)	0.85051 (14)	0.95682 (12)	0.0704 (4)	
S4	0.2296 (2)	0.90892 (14)	0.99225 (11)	0.0669 (4)	
C1	0.6117 (8)	0.2356 (5)	0.6168 (4)	0.0623 (14)	
C2	0.6779 (10)	0.3477 (6)	0.6447 (5)	0.0760 (17)	
H2	0.7043	0.3913	0.6002	0.091*	
C3	0.7044 (12)	0.3944 (7)	0.7370 (6)	0.091 (2)	
H3	0.7500	0.4694	0.7552	0.109*	
C4	0.6634 (11)	0.3302 (7)	0.8039 (5)	0.083 (2)	
H4	0.6796	0.3628	0.8666	0.100*	
C5	0.6006 (9)	0.2212 (7)	0.7784 (5)	0.0750 (18)	
H5	0.5756	0.1785	0.8238	0.090*	
C6	0.5725 (7)	0.1716 (5)	0.6842 (4)	0.0591 (13)	
C7	0.4339 (9)	0.7433 (5)	0.9440 (4)	0.0654 (15)	
C8	0.4558 (12)	0.6329 (6)	0.9180 (5)	0.086 (2)	
H8	0.5728	0.6151	0.9089	0.103*	
C9	0.3127 (16)	0.5509 (7)	0.9055 (6)	0.103 (3)	
H9	0.3319	0.4773	0.8892	0.124*	
C10	0.1387 (16)	0.5755 (7)	0.9169 (6)	0.106 (3)	
H10	0.0400	0.5183	0.9065	0.127*	
C11	0.1057 (12)	0.6862 (7)	0.9440 (5)	0.089 (2)	
H11	−0.0124	0.7031	0.9520	0.107*	
C12	0.2610 (9)	0.7703 (5)	0.9585 (4)	0.0652 (15)	
C13	0.1673 (11)	0.6604 (5)	0.6633 (4)	0.0706 (17)	
C14	0.2677 (9)	0.7576 (5)	0.7112 (4)	0.0623 (14)	
H14	0.3971	0.7642	0.7222	0.075*	
C15	0.1810 (8)	0.8458 (5)	0.7435 (4)	0.0604 (14)	
H15	0.2511	0.9111	0.7768	0.072*	
C16	−0.0127 (8)	0.8368 (5)	0.7261 (4)	0.0623 (15)	
C17	−0.1164 (10)	0.7408 (7)	0.6757 (6)	0.087 (2)	
H17	−0.2457	0.7352	0.6629	0.104*	
C18	−0.0259 (13)	0.6526 (6)	0.6441 (6)	0.096 (2)	
H18	−0.0951	0.5875	0.6098	0.115*	
C19	−0.1106 (9)	0.9293 (5)	0.7648 (5)	0.0745 (17)	
H19A	−0.1118	0.9272	0.8308	0.089*	
H19B	−0.2389	0.9173	0.7331	0.089*	
C20	0.0515 (11)	1.1170 (7)	0.8295 (5)	0.083 (2)	
H20	0.0471	1.0998	0.8890	0.100*	
C21	0.1330 (13)	1.2164 (7)	0.8205 (6)	0.092 (2)	
H21	0.1867	1.2676	0.8735	0.110*	
C22	0.1377 (10)	1.2435 (6)	0.7331 (5)	0.0774 (17)	
H22	0.1904	1.3139	0.7269	0.093*	
C23	0.0637 (7)	1.1657 (5)	0.6538 (4)	0.0599 (14)	
C24	−0.0168 (8)	1.0640 (5)	0.6672 (4)	0.0631 (14)	
H24	−0.0671	1.0098	0.6156	0.076*	
C25	0.0744 (10)	1.1905 (6)	0.5574 (5)	0.0751 (17)	
H25A	−0.0129	1.1379	0.5124	0.113*	
H25B	0.1981	1.1853	0.5443	0.113*	
H25C	0.0450	1.2643	0.5538	0.113*	
I1	0.31005 (11)	0.52863 (4)	0.61739 (4)	0.1153 (3)	
N1	−0.0234 (7)	1.0421 (4)	0.7546 (4)	0.0637 (12)	

### Atomic displacement parameters (Å^2^)

**Table:**

	*U*^11^	*U*^22^	*U*^33^	*U*^12^	*U*^13^	*U*^23^
Ni1	0.0448 (5)	0.0688 (7)	0.0653 (6)	0.0088 (5)	0.0136 (4)	0.0308 (5)
Ni2	0.0529 (6)	0.0573 (6)	0.0533 (5)	0.0025 (4)	0.0139 (4)	0.0143 (4)
S1	0.0681 (9)	0.0696 (10)	0.0701 (9)	0.0055 (7)	0.0186 (7)	0.0324 (7)
S2	0.0602 (8)	0.0762 (10)	0.0693 (9)	0.0064 (7)	0.0172 (7)	0.0335 (7)
S3	0.0628 (9)	0.0658 (10)	0.0836 (10)	0.0076 (7)	0.0165 (7)	0.0117 (8)
S4	0.0600 (9)	0.0699 (10)	0.0731 (9)	−0.0003 (7)	0.0219 (7)	0.0149 (7)
C1	0.049 (3)	0.075 (4)	0.072 (3)	0.018 (3)	0.012 (2)	0.031 (3)
C2	0.080 (4)	0.069 (4)	0.082 (4)	0.012 (3)	0.010 (3)	0.025 (3)
C3	0.099 (5)	0.072 (5)	0.098 (5)	0.009 (4)	0.004 (4)	0.015 (4)
C4	0.087 (5)	0.083 (5)	0.078 (4)	0.019 (4)	0.005 (4)	0.007 (4)
C5	0.058 (4)	0.100 (6)	0.075 (4)	0.023 (4)	0.012 (3)	0.029 (4)
C6	0.042 (3)	0.071 (4)	0.072 (3)	0.015 (2)	0.012 (2)	0.025 (3)
C7	0.076 (4)	0.062 (4)	0.055 (3)	0.004 (3)	0.004 (3)	0.011 (3)
C8	0.093 (5)	0.067 (4)	0.093 (5)	0.012 (4)	0.002 (4)	0.014 (4)
C9	0.137 (8)	0.065 (5)	0.100 (6)	−0.002 (5)	0.006 (6)	0.016 (4)
C10	0.134 (8)	0.079 (6)	0.085 (5)	−0.040 (5)	0.000 (5)	0.011 (4)
C11	0.100 (5)	0.086 (5)	0.074 (4)	−0.021 (4)	0.010 (4)	0.021 (4)
C12	0.074 (4)	0.067 (4)	0.053 (3)	−0.007 (3)	0.009 (3)	0.019 (3)
C13	0.097 (5)	0.055 (4)	0.065 (3)	0.006 (3)	0.018 (3)	0.026 (3)
C14	0.061 (3)	0.067 (4)	0.062 (3)	0.003 (3)	0.017 (3)	0.018 (3)
C15	0.063 (3)	0.065 (4)	0.056 (3)	−0.002 (3)	0.018 (2)	0.018 (3)
C16	0.054 (3)	0.069 (4)	0.074 (3)	0.002 (3)	0.015 (3)	0.041 (3)
C17	0.065 (4)	0.087 (5)	0.104 (5)	−0.011 (4)	−0.007 (4)	0.040 (4)
C18	0.119 (7)	0.062 (4)	0.098 (5)	−0.021 (4)	0.000 (5)	0.025 (4)
C19	0.059 (3)	0.070 (4)	0.108 (5)	0.008 (3)	0.031 (3)	0.045 (4)
C20	0.101 (5)	0.098 (5)	0.063 (4)	0.024 (4)	0.030 (4)	0.025 (4)
C21	0.106 (6)	0.086 (5)	0.083 (5)	0.008 (4)	0.022 (4)	0.014 (4)
C22	0.083 (4)	0.065 (4)	0.087 (4)	0.006 (3)	0.021 (3)	0.020 (3)
C23	0.050 (3)	0.068 (4)	0.075 (3)	0.020 (3)	0.022 (3)	0.033 (3)
C24	0.057 (3)	0.073 (4)	0.069 (3)	0.015 (3)	0.019 (3)	0.030 (3)
C25	0.072 (4)	0.087 (5)	0.080 (4)	0.015 (3)	0.029 (3)	0.035 (3)
I1	0.1845 (7)	0.0594 (4)	0.1157 (5)	0.0307 (4)	0.0520 (4)	0.0213 (3)
N1	0.058 (3)	0.070 (3)	0.078 (3)	0.020 (2)	0.029 (2)	0.035 (3)

### Geometric parameters (Å, °)

**Table:**

Ni1—S1^i^	2.1470 (16)	C11—H11	0.9300
Ni1—S1	2.1470 (16)	C13—C14	1.367 (9)
Ni1—S2	2.1562 (16)	C13—C18	1.391 (11)
Ni1—S2^i^	2.1562 (16)	C13—I1	2.101 (7)
Ni2—S4	2.1447 (15)	C14—C15	1.372 (9)
Ni2—S4^ii^	2.1447 (15)	C14—H14	0.9300
Ni2—S3^ii^	2.1504 (17)	C15—C16	1.393 (8)
Ni2—S3	2.1504 (17)	C15—H15	0.9300
S1—C1	1.747 (6)	C16—C17	1.376 (10)
S2—C6	1.745 (6)	C16—C19	1.492 (9)
S3—C7	1.732 (6)	C17—C18	1.387 (12)
S4—C12	1.749 (7)	C17—H17	0.9300
C1—C2	1.388 (9)	C18—H18	0.9300
C1—C6	1.390 (8)	C19—N1	1.498 (8)
C2—C3	1.364 (11)	C19—H19A	0.9700
C2—H2	0.9300	C19—H19B	0.9700
C3—C4	1.388 (10)	C20—N1	1.328 (9)
C3—H3	0.9300	C20—C21	1.331 (11)
C4—C5	1.345 (11)	C20—H20	0.9300
C4—H4	0.9300	C21—C22	1.371 (10)
C5—C6	1.399 (9)	C21—H21	0.9300
C5—H5	0.9300	C22—C23	1.388 (9)
C7—C12	1.381 (9)	C22—H22	0.9300
C7—C8	1.385 (9)	C23—C24	1.374 (8)
C8—C9	1.344 (12)	C23—C25	1.497 (8)
C8—H8	0.9300	C24—N1	1.352 (7)
C9—C10	1.369 (14)	C24—H24	0.9300
C9—H9	0.9300	C25—H25A	0.9600
C10—C11	1.414 (13)	C25—H25B	0.9600
C10—H10	0.9300	C25—H25C	0.9600
C11—C12	1.419 (9)		
			
S1^i^—Ni1—S1	180.000 (1)	C7—C12—S4	120.4 (5)
S1^i^—Ni1—S2	88.41 (6)	C11—C12—S4	119.2 (6)
S1—Ni1—S2	91.59 (6)	C14—C13—C18	119.2 (7)
S1^i^—Ni1—S2^i^	91.59 (6)	C14—C13—I1	118.9 (5)
S1—Ni1—S2^i^	88.41 (6)	C18—C13—I1	121.9 (5)
S2—Ni1—S2^i^	180.0	C13—C14—C15	121.2 (6)
S4—Ni2—S4^ii^	180.0	C13—C14—H14	119.4
S4—Ni2—S3^ii^	88.21 (6)	C15—C14—H14	119.4
S4^ii^—Ni2—S3^ii^	91.79 (6)	C14—C15—C16	119.5 (6)
S4—Ni2—S3	91.79 (6)	C14—C15—H15	120.2
S4^ii^—Ni2—S3	88.21 (6)	C16—C15—H15	120.2
S3^ii^—Ni2—S3	180.000 (1)	C17—C16—C15	120.3 (7)
C1—S1—Ni1	104.7 (2)	C17—C16—C19	119.2 (6)
C6—S2—Ni1	105.1 (2)	C15—C16—C19	120.5 (6)
C7—S3—Ni2	105.5 (2)	C16—C17—C18	119.2 (7)
C12—S4—Ni2	104.1 (2)	C16—C17—H17	120.4
C2—C1—C6	119.1 (6)	C18—C17—H17	120.4
C2—C1—S1	121.3 (5)	C17—C18—C13	120.6 (7)
C6—C1—S1	119.6 (5)	C17—C18—H18	119.7
C3—C2—C1	120.4 (7)	C13—C18—H18	119.7
C3—C2—H2	119.8	C16—C19—N1	113.7 (5)
C1—C2—H2	119.8	C16—C19—H19A	108.8
C2—C3—C4	120.2 (7)	N1—C19—H19A	108.8
C2—C3—H3	119.9	C16—C19—H19B	108.8
C4—C3—H3	119.9	N1—C19—H19B	108.8
C5—C4—C3	120.3 (7)	H19A—C19—H19B	107.7
C5—C4—H4	119.8	N1—C20—C21	120.9 (6)
C3—C4—H4	119.8	N1—C20—H20	119.5
C4—C5—C6	120.4 (7)	C21—C20—H20	119.5
C4—C5—H5	119.8	C20—C21—C22	120.1 (7)
C6—C5—H5	119.8	C20—C21—H21	120.0
C1—C6—C5	119.5 (6)	C22—C21—H21	120.0
C1—C6—S2	118.5 (5)	C21—C22—C23	120.0 (7)
C5—C6—S2	122.0 (5)	C21—C22—H22	120.0
C12—C7—C8	119.4 (6)	C23—C22—H22	120.0
C12—C7—S3	118.1 (5)	C24—C23—C22	117.5 (5)
C8—C7—S3	122.5 (6)	C24—C23—C25	121.1 (6)
C9—C8—C7	121.8 (8)	C22—C23—C25	121.4 (6)
C9—C8—H8	119.1	N1—C24—C23	120.5 (6)
C7—C8—H8	119.1	N1—C24—H24	119.7
C8—C9—C10	120.0 (8)	C23—C24—H24	119.7
C8—C9—H9	120.0	C23—C25—H25A	109.5
C10—C9—H9	120.0	C23—C25—H25B	109.5
C9—C10—C11	121.4 (8)	H25A—C25—H25B	109.5
C9—C10—H10	119.3	C23—C25—H25C	109.5
C11—C10—H10	119.3	H25A—C25—H25C	109.5
C10—C11—C12	116.9 (8)	H25B—C25—H25C	109.5
C10—C11—H11	121.5	C20—N1—C24	121.0 (6)
C12—C11—H11	121.5	C20—N1—C19	120.9 (5)
C7—C12—C11	120.4 (7)	C24—N1—C19	118.1 (6)
			
S2—Ni1—S1—C1	6.0 (2)	S3—C7—C12—C11	176.6 (5)
S2^i^—Ni1—S1—C1	−174.0 (2)	C8—C7—C12—S4	179.5 (5)
S1^i^—Ni1—S2—C6	173.59 (19)	S3—C7—C12—S4	−1.8 (7)
S1—Ni1—S2—C6	−6.41 (19)	C10—C11—C12—C7	1.6 (9)
S4—Ni2—S3—C7	−1.8 (2)	C10—C11—C12—S4	−179.9 (5)
S4^ii^—Ni2—S3—C7	178.2 (2)	Ni2—S4—C12—C7	0.2 (5)
S3^ii^—Ni2—S4—C12	−178.97 (19)	Ni2—S4—C12—C11	−178.3 (4)
S3—Ni2—S4—C12	1.03 (19)	C18—C13—C14—C15	2.4 (9)
Ni1—S1—C1—C2	175.1 (5)	I1—C13—C14—C15	−179.3 (4)
Ni1—S1—C1—C6	−4.3 (5)	C13—C14—C15—C16	−0.9 (8)
C6—C1—C2—C3	0.1 (10)	C14—C15—C16—C17	−1.0 (8)
S1—C1—C2—C3	−179.3 (6)	C14—C15—C16—C19	176.8 (5)
C1—C2—C3—C4	−0.7 (12)	C15—C16—C17—C18	1.3 (10)
C2—C3—C4—C5	1.2 (12)	C19—C16—C17—C18	−176.5 (6)
C3—C4—C5—C6	−1.2 (11)	C16—C17—C18—C13	0.2 (11)
C2—C1—C6—C5	−0.1 (8)	C14—C13—C18—C17	−2.0 (10)
S1—C1—C6—C5	179.3 (4)	I1—C13—C18—C17	179.7 (5)
C2—C1—C6—S2	179.7 (5)	C17—C16—C19—N1	−138.2 (6)
S1—C1—C6—S2	−0.9 (6)	C15—C16—C19—N1	44.0 (8)
C4—C5—C6—C1	0.6 (9)	N1—C20—C21—C22	1.1 (13)
C4—C5—C6—S2	−179.1 (5)	C20—C21—C22—C23	−2.4 (12)
Ni1—S2—C6—C1	5.6 (5)	C21—C22—C23—C24	1.7 (10)
Ni1—S2—C6—C5	−174.6 (4)	C21—C22—C23—C25	−177.1 (7)
Ni2—S3—C7—C12	2.5 (5)	C22—C23—C24—N1	0.3 (8)
Ni2—S3—C7—C8	−178.9 (5)	C25—C23—C24—N1	179.1 (5)
C12—C7—C8—C9	0.6 (10)	C21—C20—N1—C24	1.0 (11)
S3—C7—C8—C9	−178.0 (6)	C21—C20—N1—C19	178.4 (7)
C7—C8—C9—C10	1.2 (12)	C23—C24—N1—C20	−1.7 (8)
C8—C9—C10—C11	−1.6 (13)	C23—C24—N1—C19	−179.2 (5)
C9—C10—C11—C12	0.3 (11)	C16—C19—N1—C20	−115.7 (7)
C8—C7—C12—C11	−2.0 (9)	C16—C19—N1—C24	61.8 (7)

Symmetry codes: (i) −*x*+1, −*y*, −*z*+1; (ii) −*x*+1, −*y*+2, −*z*+2.

### Hydrogen-bond geometry (Å, °)

**Table:**

*D*—H···*A*	*D*—H	H···*A*	*D*···*A*	*D*—H···*A*
C19—H19B···S2^iii^	0.97	2.81	3.613 (7)	141
C20—H20···S4^iv^	0.93	2.86	3.580 (8)	135

Symmetry codes: (iii) *x*−1, *y*+1, *z*; (iv) −*x*, −*y*+2, −*z*+2.
